# Controversies in Targeted Therapy of Adult T Cell Leukemia/Lymphoma: ON Target or OFF Target Effects?

**DOI:** 10.3390/v3060750

**Published:** 2011-06-14

**Authors:** Rihab Nasr, Hiba El Hajj, Youmna Kfoury, Hugues de Thé, Olivier Hermine, Ali Bazarbachi

**Affiliations:** 1 Department of Anatomy, Cell Biology and Physiological Sciences, Faculty of Medicine, American University of Beirut, Beirut 1107 2020, Lebanon; 2 Department of Internal Medicine, Faculty of Medicine, American University of Beirut, Beirut 1107 2020, Lebanon; E-Mails: he21@aub.edu.lb (H.E.H.); yk02@aub.edu.lb (Y.K.); 3 Service de Biochimie, Equipe labellisée, Ligue Nationale Contre le Cancer, Centre National de la Recherche Scientifique, Institut National de la Santé et de la Recherche Médicale, Université Paris Diderot Unité Mixte de Recherche 7212, Unité 944, Hôpital Saint Louis, 75475 Paris, Cedex 10, France; E-Mail: dethe@univ-paris-diderot.fr (H.d.T.); 4 Service d’hématologie, Equipe labellisée, Ligue Nationale Contre le Cancer, Centre National de la Recherche Scientifique, Université Paris Descartes Unité Mixte de Recherche 8147, Hôpital Necker-Enfants Malades, 75015 Paris, Cedex 15, France; E-Mail: hermine@nck.aphp.fr (O.H.)

**Keywords:** adult T cell leukemia, HTLV-I, Tax, AZT, arsenic trioxide, interferon

## Abstract

Adult T cell leukemia/lymphoma (ATL) represents an ideal model for targeted therapy because of intrinsic chemo-resistance of ATL cells and the presence of two well identified targets: the HTLV-I retrovirus and the viral oncoprotein Tax. The combination of zidovudine (AZT) and interferon-alpha (IFN) has a dramatic impact on survival of ATL patients. Although the mechanism of action remains unclear, arguments in favor or against a direct antiviral effect will be discussed. Yet, most patients relapse and alternative therapies are mandatory. IFN and arsenic trioxide induce Tax proteolysis, synergize to induce apoptosis in ATL cells and cure Tax-driven ATL in mice through specific targeting of leukemia initiating cell activity. These results provide a biological basis for the clinical success of arsenic/IFN/AZT therapy in ATL patients and suggest that both extinction of viral replication (AZT) and Tax degradation (arsenic/IFN) are needed to cure ATL.

## 1. The First Potential Target: HTLV-I Retrovirus

The human T cell lymphotropic virus type I (HTLV-I) infects approximately 20 million individuals worldwide [1]. Endemic areas include Japan, the Caribbean, inter-tropical and South Africa, South America, some regions of Romania and the Middle East [2–4]. HTLV-I infection causes two major types of diseases: HTLV-associated myelopathy/tropical spastic paraparesis (HAM/TSP), an incapacitating inflammatory disease of the central nervous system, and adult T-cell leukemia/lymphoma (ATL), a distinct peripheral T-cell malignancy [5–7]. In addition, HTLV-I infection also results in immune suppression resulting in increased risk for coinfections such as Strongyloides Stercoralis [8].

ATL develops in a small percentage (4%) of HTLV-I-infected individuals after a long period of clinical latency following viral infection [9]. The late onset of ATL indicates that HTLV-I infection is not sufficient to induce T-cell transformation and that additional genetic mutations favoring the leukemogenic process are needed before the onset of ATL [10]. Yet, ATL is characterized by the monoclonal or oligoclonal integration of HTLV-I provirus in the tumor cells [11]. Typical ATL cells are characterized by unusual morphology with a lobulated nucleus, known as “flower cells” [12]. These malignant lymphocytes are activated CD4+ CD25+ T cells with increased expression of the alpha chain of the interleukin-2 receptor [13]. In addition to the classical structural genes required for retroviral replication, the HTLV-1 genome encodes a series of auxiliary and regulatory proteins such as the viral transcriptional activator Tax and the HTLV-1 bZIP factor gene (HBZ), a viral protein encoded from the 3′ LTR in the complementary strand of the proviral genome [14,15]. Both Tax and HBZ expression is linked to different stages of HTLV-I pathogenesis [16,17].

HTLV-1 infects CD4+ and CD8+ T lymphocytes and can also efficiently infect dendritic cells [17]. The ubiquitous glucose transporter 1 and the neuropilin 1 were identified as members of the HTLV-I receptor complex [18–20]. Moreover, surface heparan sulfate proteoglycans were shown to be required for efficient virus entry [21,22]. Clonal expansion of HTLV-infected cells mostly relies on the promotion of cycling CD4+ T cells. Indeed, the proliferation of infected CD4+ cells, correlates with the level of Tax expression, resulting in accumulation of cellular defects [23,24].

## 2. The Second Potential Target: The Viral Oncoprotein Tax

In addition to its effects on the viral LTR, Tax has pleiotropic cellular functions [1,25–27]. It stimulates the transcription of several cellular genes through activation of critical transcription factors such as nuclear factor kappa B (NF-κB) [28–33], cyclic AMP response element-binding protein (CREB), serum responsive factor (SRF), and activated protein 1 (AP-1) [34–37]. Recent findings indicate that Tax post-translational modifications, namely ubiquitylation and SUMOylation, are critical for the constitutive activation of the NF-κB pathway [38–40].

Tax also represses the expression of cellular genes such as DNA polymerase β, cyclin A and transforming growth factor β [41–43]. Moreover, Tax is involved in the regulation of apoptosis through the activation of apoptosis-suppressing genes such as Bcl-XL [44]. Tax inhibits the function of tumor suppressor proteins such as p53 and p16, interferes with cell cycle checkpoint control and enhances the accumulation of mutations in HTLV-I infected cells through the repression of DNA repair [45–47]. Recent observations suggested that Tax also modulates the micro-RNA environment thereby adding another level of complexity to its cellular functions [48]. Altogether, these multiple activities of Tax cooperate to promote infected T-cell proliferation, generate cellular defects and lead to subsequent transformation.

The oncogenic potential of Tax has been extensively studied. Tax was shown to transform a rat fibroblast cell line and immortalize primary T cells *in vitro* [49,50]. Importantly, *tax*-transgenic mice that express Tax under the control of the Lck-proximal promoter, which restricts Tax expression to immature thymocytes, develop a leukemia/lymphoma with striking ATL features that recapitulates the human disease [51]. Moreover, reconstitution of NOD-SCID mice with HTLV-1 *tax*-transduced human CD34+ cells resulted in the development of CD4+ lymphomas [52]. Altogether, these findings support an important role for Tax in the proliferation of infected cells, formally demonstrating that Tax alone can initiate ATL. It is noteworthy to mention however, that ATL occurs in less than 5% of HTLV-I infected individuals [9]. Whether continuous Tax expression is required for maintenance of the transformed phenotype is still controversial.

## 3. Is Tax Really a Target in ATL?

Based on its multiple effects, Tax was proposed to be an oncogene in ATL, but the fact that Tax protein expression is frequently undetectable *in vivo* has led to significant controversies. Indeed, viral expression is extremely limited *in vivo* and Tax protein expression is undetectable in the vast majority of ATL cells. Importantly, Tax expression has two major influences on HTLV-1 infected cells: first, as stated above, Tax recruits CD4+ T cells into the cell cycle and drives their proliferation and clonal expansion [23,24]; on the other hand, Tax is highly immunogenic and its expression induces an immune response that generates cytotoxic T lymphocytes (CTL) chiefly directed against this oncoprotein. Therefore, in order to escape this CTL-mediated lysis and to maintain viral persistence *in vivo*, HTLV-I infected cells and ATL cells frequently reduce the expression of Tax by several mechanisms. For example, the loss of 5′-LTR which is essential for viral transcription, and the nonsense/mis-sense mutations of the *tax* gene, will both reduce viral expression. In addition, epigenetic changes in the 5′-LTR may also silence the transcription of viral genes [53–56]. Moreover, the HBZ protein, which is constitutively expressed in all ATL and HTLV-I infected cells, was shown to reduce Tax expression and Tax-mediated viral transcription [15,57]. Furthermore, HTLV-I auxiliary proteins such as p30 may play a critical role in slowing the replication of HTLV-I infected cells, possibly through LTR silencing [58,59]. Interestingly, mutations in Tax MHC class I recognition site also results in an escape of infected cells from immune recognition [54]. Therefore, it could be speculated that this repression in Tax expression is essential to protect infected cells from immune response and to maintain the virus *in vivo*. However, recent studies suggested that a loss of Tax expression does not only allow transformed T cells to evade CTL killing but also prevents Tax-induced mitotic aberrations that are detrimental to cell proliferation and therefore, to stabilize the karyotype of infected T cells [60].

On the other hand, the lack of HTLV-I expression *in vivo* may be also explained by the existence of a viral reservoir, outside the blood compartment or by a transient or low-level expression of the Tax protein undetectable by Western blot. Indeed, HTLV-1 infection of dendritic cells could provide a reservoir for HTLV-1 infection [61]. These dendritic cells likely play a central role in HTLV-I transmission, dissemination and persistence *in vivo*. The presence of other hidden viral reservoirs may be also suggested. In that sense, the recapitulation of a CD4+ T-cell lymphoma in humanized-NOD/SCID mice injected with CD34+ cells transduced with Tax [52] suggests that hematopoietic stem cells provide a viral reservoir *in vivo*.

Interestingly, the level of Tax mRNA in murine ATLs derived from Tax transgenics is strikingly similar to the one found in human ATLs but 1000-fold less than in HTLV-I infected cell-lines [62]. This dramatic up-regulation of Tax in laboratory cell-lines may reflect some adaptation to *ex vivo* culture. In that sense, Tax expression is rapidly induced in *ex vivo* culture of primary HTLV-I infected lymphocytes. This strongly suggests that the microenvironment of ATL cells *in vivo* has an inhibitory effect on Tax expression [63]. Moreover, that Tax protein is not detected following transplantation of murine ATL cells in severely immunodeficient mice, indicates that the down-regulation of Tax expression is not only due to the immune pressure against cells expressing this oncoprotein. Alternatively, this low level of Tax expression may be explained by the potential cellular toxicity of high levels of Tax oncoprotein. Indeed, in previous *in vitro* studies, high levels of Tax had proapoptotic and anti-proliferative effects, whereas lower Tax levels stimulated cell cycle progression (reviewed in [64]). Moreover, previous reports demonstrated that Tax readily commits immortalized and transformed cells to senescence [65]. Therefore, one may suggest that ATL cells reduce *in vivo* Tax expression in order to escape Tax-induced cell death. Another explanation for this viral latency *in vivo* is a rapid destruction by the immune system of cells expressing HTLV-I proteins at the cell surface. Interestingly, HTLV-1 infected CD8+ T cells are themselves the subject of killing by virus specific CTL. Indeed, this fratricide among virus-specific CTLs could impair the immune control of HTLV-I contributing therefore to viral persistence *in vivo* [66].

Taking all these observations into account, it seems that there is dynamic equilibrium between viral persistence and the immune response that regulates the number of Tax-expressing cells and that Tax is indispensable for the transformation process induced in HTLV-I infected cells. Interestingly, using a specific technique that permits the quantification of lymphocyte turnover in humans *in vivo*, Asquith *et al*. provided evidence for continuous low-level of HTLV-1 expression *in vivo* [67]. They indeed demonstrated that Tax expression is correlated with CD4+CD45RO+ T cell proliferation therefore suggesting persistent viral gene expression *in vivo*. Increased CD4+CD45RO+ cell proliferation would also increase the probability of acquiring mutations that could increase the risk of malignant transformation and the development of ATL.

Although HBZ, that allows infected cells to evade an immune response through prevention of Tax overexpression [15], is needed to promote infected cell proliferation and persistence in the late steps of infection, it is still unknown whether, once cells are transformed, continuous Tax expression is required for maintenance of the leukemic state. HBZ transgenics develop lymphoid tumors [68]. However, Tax transgenics and ATL cells are characterized by constitutive NF-κB activation [62], whereas NF-κB is not activated in malignant cells from HBZ transgenics. These indirect arguments suggest that the maintenance of the ATL phenotype requires continuous Tax expression.

## 4. Conventional Therapy for ATL

The diversity in prognosis and clinical features of patients with ATL led to the Shimoyama classification of this disease into four clinical subtypes: acute, lymphoma, chronic and smoldering forms [69]. This classification is largely based on the extent of systemic leukemia, hypercalcemia and organ involvement. The chronic and smoldering subtypes are considered indolent, but eventually have poor long-term survival [70]. The acute and lymphoma forms generally have a worse prognosis mainly due to their resistance to the conventional chemotherapies, a large tumor burden, hypercalcemia, and/or frequent infectious complications as a result of a profound T-cell immunodeficiency [71,72]. Until recently, ATL was a nearly incurable disease with an overall median survival of less than one year in the aggressive forms. Complex combinations of chemotherapy regimen, such as the LSG15 protocol, resulted in an improved response rate particularly in the lymphoma subtype, however, long-term survival remains dismal [73]. Promising results were reported in selected patients undergoing allogeneic stem cell transplantation, possibly reflecting a graft-*versus*-ATL effect (reviewed in [74]). Yet, this procedure is only considered in eligible young ATL patients with available donors, with an extremely careful attention because of risk of opportunistic infections and significant transplant related mortality. Thus alternative therapies are still mandatory.

## 5. Targeted Therapies for ATL

### 5.1. Breakthrough in ATL Therapy: The Combination of Zidovudine and Interferon-Alpha

An important advance in the treatment of ATL was initially reported in two preliminary phase II studies using the combination of an anti-retroviral agent zidovudine (AZT) and interferon-alpha (IFN). These initial reports accumulated promising evidence for the effectiveness of antiviral therapy in the treatment of ATL and suggested that this combination should be further evaluated. Hermine *et al.* demonstrated that the combination of AZT and IFN induces a rapid and durable response in five ATL patients [75]. Gill *et al.* showed that 11 out of 19 patients with acute or lymphoma ATL achieved major responses with the combination therapy (five complete remissions and six partial remissions) [76]. However, almost half of these patients had either relapsed or refractory disease. Bazarbachi *et al.* showed that the combination of AZT and IFN exhibited a high response rate and prolonged survival of previously untreated ATL patients [77]. However, in 2001, White *et al*. reported lower response rate in heavily pretreated ATL patients. Subsequently, numerous small studies confirmed the promising results achieved with AZT/IFN combination [78]. A British study reported that two-thirds of 15 ATL patients achieved and/or maintained a response to the combination of IFN and AZT [79]. One year later, the French ATL group published the results of a prospective phase II clinical trial with the use of AZT/IFN combination as first line therapy or after initial chemotherapy in 18 patients with aggressive ATL (acute and lymphoma subtypes) [80]. The initially encouraging results of the combination AZT/IFN were confirmed in this phase II study with an overall response rate of 76%. Interestingly, the response rate was very high in previously untreated patients with acute ATL, since two CR and four PR (>95%) were observed in six out of seven patients. Moreover, these studies suggested that the synergistic effect between the two antiviral drugs AZT and IFN seems to be critical for a successful response. However, despite the benefit observed with this regimen, relapse occurred in most patients, demonstrating that this combination did not cure ATL. Interestingly, the reintroduction of AZT/IFN induced second remissions in relapsed patients that were off therapy demonstrating that continuous maintenance therapy with AZT/IFN is mandatory. In Iran, a recent study showed that AZT/IFN treatment induced a high response rate and potentially prolonged survival with minimal side effects [81]. In a recent prospective phase II study in the USA, 19 ATL patients received infusional chemotherapy (EPOCH regimen) until maximal response, followed by antiviral therapy with daily AZT, lamivudine, and IFN. However, because of disease progression, only six patients received antiviral therapy [82]. Finally, a retrospective analysis in Martinique revealed a significant increase in ATL survival after 1995, when antiviral therapy was introduced [83].

Although these multiple small studies demonstrated that AZT/IFN therapy is potentially beneficial for ATL, the impact of this antiviral combination on the long-term survival of ATL patients was still undetermined, until recently when we reported the results of a large worldwide meta-analysis on the use of IFN and AZT for ATL. In this analysis, we studied medical records of 254 patients with ATL who were treated in various countries (United States, United Kingdom, Martinique, and continental France). The combination of AZT and IFN had a dramatic impact on the survival of ATL patients, with a five-year overall survival (OS) of 46% for 75 patients who received first line antiviral therapy alone *versus* 14% for 132 patients who received first line chemotherapy with or without antiviral maintenance. In acute ATL, first-line antiviral therapy alone resulted in a significant survival advantage (five-year OS of 28%) as compared with first-line chemotherapy with or without maintenance antiviral therapy (five-year OS of 10%). Achievement of CR with antiviral therapy in acute ATL resulted in 82% five-year survival. Importantly, patients with ATL lymphoma did not benefit from the combination of AZT and IFN indicating that standard chemotherapy should remain the mainstay of treatment for this subtype. Finally, first-line AZT/IFN therapy in chronic- and smoldering-type ATL resulted in 100% OS at a median follow-up time of five years [84]. These are very promising results specifically when compared to the results reported in a recently published Japanese study in which the five-year OS was as low as 47% in patients with indolent ATL that were managed by a watchful waiting policy until disease progression or by chemotherapy [85].

Therefore, the results of this meta-analysis led to the conclusion that the treatment of ATL using the combination of AZT and IFN induces high response and CR rates and significantly prolongs survival particularly in acute, chronic, and smoldering types of ATL, but not in lymphoma ATL. Accordingly, this highly beneficial combination should be considered as the gold standard first-line therapy in leukemic subtypes of ATL. This combination is mostly effective as initial therapy but less effective after prior treatment with chemotherapy, most likely due to the selection of a tumor clone with mutated p53 when first line chemotherapy is used.

In summary, the combination of AZT and IFN has clearly changed the natural history of ATL through achievement of a significantly improved long-term survival in patients with smoldering and chronic ATL as well as a subset of patients with acute ATL and wild type p53 status. Unfortunately, most patients eventually relapsed, suggesting that AZT/IFN could not cure ATL and that new therapeutic approaches are still needed.

### 5.2. The AZT/IFN Combination: Is it an Antiviral Therapy?

The success of this potentially antiretroviral approach in the treatment of ATL raises important questions on the mechanism of action of this combination. Zidovudine, formerly called azidothymidine (AZT), is an antiviral pyrimidine nucleoside analogue that inhibits the reverse transcriptase of the human immunodeficiency virus (HIV) and the HTLV-1 virus [86]. AZT does not have a general antineoplastic activity because it is a poor substrate for mammalian DNA polymerase-α [71]. On the other hand, interferons (IFN) are a family of glycoproteins whose mechanism of action is complex and not well understood. IFN have potent broad-spectrum antiviral effect. They activate several interferon-responsive genes that have antiviral activity. IFN have also an antiproliferative effect and can inhibit the cell growth of several tumor cells. Moreover, IFN have an immunomodulatory effect and may therefore stimulate the immune system. Furthermore, IFN activate several immune cells like natural killer cells and macrophages, increase antigen presentation to lymphocytes, and induce the resistance of host cells to viral infection helping in slowing down tumor cell growth [87]. Finally, a recent report demonstrated that plasmacytoid dendritic cells respond to cell-free HTLV-1 by producing high levels of IFN and by mobilizing TRAIL on cell surface after TLR7 triggering [88].

Several arguments are in favor of an antiviral effect of the AZT/IFN combination. In a rabbit model of ATL, AZT inhibited HTLV-I proviral load [89]. Moreover, AZT inhibited the transmission of HTLV-I to adult peripheral blood mononuclear cells *in vitro* and blocked the transformation of these normal blood lymphocytes when co-cultured with an HTLV-I transformed cell line [90]. Recently, it was shown that a combination of AZT with an inhibitor of histone deacetylases decreases the proviral load and increases virus-specific cytotoxic T-cell population in a series of baboons naturally infected with simian T-lymphotropic virus type 1 (STLV-1) confirming the antiviral effect of AZT [91]. Recent studies demonstrated that IFN inhibited HTLV-I virus assembly by preventing targeting of viral Gag proteins to the rafts in the plasma membrane, demonstrating its antiviral effect [92]. Among its multiple biological effects, IFN has an immunomodulatory effect that should be also considered. As stated before, HTLV-I infected individuals are known to have a strong CTL response against cells that express the Tax protein [93] and IFN may induce the expression of viral antigens or major histocompatibility complex molecules to enhance the CTL response against the HTLV-I infected cells. Interestingly, recent reports indicate that HTLV-1 evades type I IFN antiviral signaling by inducing the suppressor of cytokine signaling 1 (SOCS1). Of particular interest, SOCS1 was upregulated in HAM/TSP patients and asymptomatic carriers but not in ATL [94].

On the other hand, a potentially cytotoxic effect of the AZT/IFN combination on leukemic cells cannot be excluded. The addition of IFN and AZT inhibited cell growth of myeloid leukemia cell lines, *in vitro* [95]. However, we have reported compelling evidence that the therapeutic effect of AZT/IFN is not through a direct cytotoxic effect of these drugs on the ATL leukemic cells. Indeed, AZT, IFN and their combination failed to induce any significant inhibition of cell proliferation, cell cycle arrest or apoptosis of HTLV-I positive cell lines even in long-term culture. Furthermore, we observed no *in vitro* cytotoxic effect of the AZT/IFN combination on fresh leukemic cells derived from an acute ATL patient at diagnosis despite achievement of *in vivo* complete remission using the same therapy [96].

Datta *et al.* demonstrated that treatment of HTLV-1 infected cells with AZT leads to telomere shortening, induction of cellular senescence and reactivation of p53 functional activities. They also showed a direct correlation between patient’s response to AZT/IFN and p53 status [97]. Accordingly, the analysis of p53 sequence in the malignant cells before the beginning of AZT/IFN treatment could be highly beneficial to predict disease outcome. Patients with mutated p53 that may not respond to AZT/IFN treatment should be treated with other alternative ATL treatments. However, the lack of efficacy of AZT outside the settings of virus-associated malignancies argues against the hypothesis that telomerase and p53 are the main targets of this drug. In that sense, the combination AZT/IFN is not effective in Tax-transgenic murine ATL [98]. In this model, Tax expression is under the lck promoter and there is no viral replication.

One may argue that the HTLV-I virus does not replicate in ATL cells. To reconciliate these contradictory findings, we can hypothesize that the AZT/IFN combination targets viral replication outside ATL cells, such as in dendritic cells or other cell types in which a dynamic continuous viral replication occurs. In that case, this viral replication can provide the microenvironment that is mandatory for survival of ATL cells through direct cell-cell communication or paracrine stimulation through secreted Tax or various cytokines/chemokines as reported for chronic lymphocytic leukemia [99]. This explains the impossibility to culture ATL cells *in vitro* and the effectiveness of the AZT/IFN combination *in vivo* but not *in vitro.* Similarly, the AZT/IFN combination, through inhibition of *de novo* infection of circulating CD8+ lymphocytes, that are subject to killing by virus specific CTL [66], may protect the immune system from the fratricide among these virus-specific CTLs, allowing therefore disease control by the immune system.

Finally, from a philosophical point of view, it is interesting to note that the only malignancy in which the antiviral drug AZT is highly effective is ATL which is the only human malignancy secondary to a retroviral infection. In summary, even though the mechanism of action of AZT/IFN has not been fully elucidated yet, multiple clinical trials have clearly shown that the combination of AZT and IFN improves the survival of ATL patients but cannot cure ATL.

### 5.3. Targeting ATL Leukemia Initiating Activity: The Combination of Arsenic Trioxide and IFN

Besides the combination of AZT and IFN, one of the most promising targeted therapies against ATL is the combination of IFN and arsenic trioxide, a very effective treatment against acute promyelocytic leukemia (APL) [100,101]. In fact, it all started around ten years ago when we first demonstrated that the combination of arsenic and IFN act synergistically to induce cell cycle arrest and apoptosis, specifically in HTLV-I transformed cells and fresh ATL leukemia cells, providing a biological basis for arsenic/IFN treatment in ATL [102,103]. At the molecular level, we reported that the arsenic/IFN combination kills ATL derived cells through rapid reversal of the constitutive activation of NF-κB and delayed shut down of cell cycle-regulated genes secondary to Tax degradation by the proteasome that was concomitant with cell death induction, thus indicating the importance of continuous Tax expression for ATL cell survival [104,105]. These results prompted us to investigate the efficacy of this combination in ATL patients. Therefore, we initiated a phase II clinical trial of arsenic/IFN in seven patients with relapsed or refractory ATL after AZT/IFN/chemotherapy. Four patients responded with one complete remission and three partial remissions [106]. One patient was still alive and free of disease five years later. Despite the poor prognosis of the selected patients enrolled in this study, we demonstrated that treatment with arsenic/IFN is feasible and exhibits anti-leukemic effect. Later in 2007, Ishitsuka *et al*. reported that arsenic/IFN combination yielded clinical results in two moderately-aggressive ATL patients [107].

The efficacy and the toxicity of this combined treatment were also evaluated in HTLV-1-infected squirrel monkeys and HTLV-1 infected cell lines derived from it. It was shown that arsenic/IFN can induce growth arrest in HTLV-1-transformed monkey T-cell lines *in vitro* and reduce the total number of circulating HTLV-1 flower cells in the infected monkeys with chronic ATL-like disease [108].

We recently reported the results of a prospective phase II trial in which arsenic was added to the combination of AZT and IFN in newly diagnosed chronic ATL. Interestingly, all 10 patients enrolled in this trial responded, including seven patients who achieved CR, two patients who achieved very good partial response (clinical and biologic CR except for the presence of more than 5% atypical lymphocytes on peripheral blood smear), and one patient achieved partial response. Side effects were moderate and no relapse was noted at the time of reporting [109]. These encouraging results suggest that the triple combination of arsenic, AZT and IFN is a promising approach that should be further evaluated in the first line therapy of ATL.

To investigate the molecular mechanism of therapeutic action *in vivo*, we used *Tax* transgenic mice that develop a disease with striking ATL features including circulating ATL like flower cells, massive organ infiltration, high LDH, malignant hypercalcemia and constitutive NF-kB activation in the leukemic cells [51]. We recently reported that the combination of arsenic trioxide and IFN cures Tax-driven murine ATLs through selective targeting of leukemia initiating cell (LIC) activity. Indeed, unexpectedly, ATL cells did not respond to arsenic and IFN by apoptosis and/or cell cycle arrest *in vivo* and therefore this regimen does not induce rapid tumor regression or massive cell death in treated animals. On the other hand, we showed that this combination therapy used in primary mice immediately reduces leukemia transplantation into untreated secondary recipients and totally abrogates leukemia transplantation into untreated tertiary recipients. In other words, the primary tumor continues to grow and only exhausts much later, due to the specific targeting of LIC activity. Adding the proteasome inhibitor bortezomib essentially blocks the degradation of Tax triggered by the arsenic/IFN combination, and eliminates the enhancement of survival in secondary and tertiary recipients. This reversal of ATL LIC eradication by proteasome inhibition is a significant indication that ATL cells are addicted to continuous Tax expression for their LIC activity (stemness) but not for their short-term tumor growth [62].

### 5.4. Are We Really Targeting Tax?

Proteasome-mediated degradation of Tax and targeting of ATL LIC activity by arsenic/IFN combination is reminiscent of the proteasome-mediated degradation of PML-RAR and targeting of APL LIC by arsenic in APL [62,101]. But does LIC eradication by arsenic/IFN really rely on Tax degradation?

As previously described, microarray analysis of the gene expression profile shows that both arsenic and arsenic/IFN specifically block the transcription of NF-κB dependent genes which results in the reversal of NF-κB activation [104]. Although activation of the NF-κB pathway has been reported to play a crucial role in the proliferation of ATL leukemia cells, its sole inhibition by arsenic alone is not enough to explain the *in vivo* effects of the combination. Moreover, we have also demonstrated that bortezomib, triggers NF-κB inhibition in ATL cells [110]. However, neither arsenic alone nor bortezomib alone are sufficient to eradicate ATL LIC activity [62].

One may argue that there is low or even no expression of Tax *in vivo*, so how to explain the *in vivo* efficacy of the arsenic/IFN combination? As discussed previously, the issue of Tax expression in HTLV infected cells or in ATL cells is still controversial. Strong evidence in favor of Tax expression is the existence of Tax specific antibodies in the serum of ATL patients and the presence of the Tax-specific CD8+ T cells recently demonstrated in ATL patients (reviewed in [111,112]). Moreover, although Tax is not detectable in freshly harvested cells, it is up regulated once the cells are cultured *ex vivo* [113] strongly suggesting that Tax is expressed in at least some ATL cells. In that sense, a recent report indicate that human and mouse stromal cells, such as epithelial cells and fibroblasts, suppressed HTLV-1 expression in ATL and non-ATL HTLV-1-infected cells. When infected cells were reisolated from the cocultures, viral expression was restored to the original level over the following 48 h. Spontaneous induction of HTLV-1 expression in primary ATL cells in the first 24 h of culture was also inhibited by coculture with stromal cells. Indeed, coculture of HTLV-1-infected cells and stromal cells induced type I IFN responses. These findings indicate that the innate immune system suppresses HTLV-1 expression *in vivo*, at least through type I IFN [63].

Although other viral factors can promote pathogenesis such as HBZ, tax-transgenic mice develop a disease that strikingly recapitulates human ATL [68]. Conversely, although HBZ transgenics develop lymphoid tumors, these transformed cells are devoted from NF-kB activation and hence are different from ATL cells. Moreover, in Tax-transgenic mice, although tax mRNA is detectable, there is no constitutive detectable expression of the Tax protein in the transformed T cells. Interestingly, the level of tax expression in these transgenic mice is very similar to the level found in ATL patients and 1000-times less than that in cell lines [62] indicating that high levels of Tax may be toxic *in vivo* and only low levels are needed for transformation.

The mechanism as to how viral expression is maintained *in vivo* in this latent form is not understood. One possible explanation is the existence of a viral reservoir *in vivo*, which may involve dendritic cells. In that sense, Jones *et al*., 2008 [61] showed that HTLV-I can infect dendritic cells that can efficiently transfer virus to autologous primary CD4+ cells indicating that dendritic cells have a central role in HTLV-I transmission, dissemination and persistence *in vivo*.

Overall, based on the high specificity of the IFN/arsenic combination to both HTLV-I-infected cells and Tax-driven murine ATL, it is most likely that therapy-induced loss of the driving oncogene underlies therapy responses. Hence, we suggest the existence of a hierarchy among genetic defects in ATL: Tax clearly initiates the disease while HBZ and other secondary somatic mutations in cellular genes favor its progression. As for many other types of leukemia, secondary events are unlikely to be ideal targets for curative therapies whereas earlier genetic events are the most important for the maintenance of the leukemic clone proliferation and survival. Our results suggest that Tax is required for both initiation of ATL and for the maintenance of the ATL LIC activity (stemness) but is dispensable for short-term growth. Hence, the combination of arsenic and IFN cures ATL through proteasome-dependent oncoprotein degradation resulting in selective eradication of the ATL LIC activity.

## 6. Conclusion

On the basis of these findings, the triple combination of AZT, IFN and arsenic represents an appealing first-line therapy of ATL. In that sense, three out of six chronic ATL patients who stopped therapy after achieving complete remission with the triple combination of AZT, IFN and arsenic, remained in CR after 18 months, an unprecedented finding. Furthermore, the use of the IFN/arsenic combination as a consolidation therapy after chemotherapy allowed very long lasting remissions [114], suggesting that it can efficiently target ATL LIC activity. However, these findings are still preliminary and need confirmation in larger studies before they can be routinely used in ATL therapy. In conclusion, our results raise hopes that extinction of viral replication (AZT/IFN) and Tax degradation with subsequent eradication of LIC activity (arsenic/IFN) may eradicate the disease resulting in ATL cure rather than long-term disease control.
